# Species–environment interactions changed by introduced herbivores in an oceanic high-mountain ecosystem

**DOI:** 10.1093/aobpla/plw091

**Published:** 2017-01-05

**Authors:** Jaume Seguí, Marta López-Darias, Antonio J. Pérez, Manuel Nogales, Anna Traveset

**Affiliations:** 1 Department of Global Change, Mediterranean Institute for Advanced Studies (CSIC-UIB), C/Miquel Marquès 21, 07190-Esporles, Mallorca, Balearic Islands, Spain; 2 Department of Agrobiology and the Environment, Institute of Natural Products and Agrobiology (CSIC), C/Astrofísico Fco. Sánchez 3, 38206-La Laguna, Tenerife, Canary Islands, Spain

**Keywords:** Autofertility, Canary Islands, elevational gradient, herbivory, pollen limitation, rabbits, *Viola cheiranthifolia*

## Abstract

Summit areas of oceanic islands constitute some of the most isolated ecosystems on earth, highly vulnerable to climate change and introduced species. Within the unique high-elevation communities of Tenerife (Canary Islands), reproductive success and thus the long-term survival of the species may depend on environmental suitability as well as threat by introduced herbivores. By experimentally modifying the endemic and vulnerable species *Viola cheiranthifolia* along its entire altitudinal occurrence range, we studied plant performance, autofertility, pollen limitation and visitation rate and the interactive effect of grazing by non-native rabbits on them. We assessed the grazing effects by recording (i) the proportion of consumed plants and flowers along the gradient, (ii) comparing fitness traits of herbivore-excluded plants along the gradient, and (iii) comparing fitness traits, autofertility and pollen limitation between plants excluded from herbivores with unexcluded plants at the same locality. Our results showed that *V. cheiranthifolia* performance is mainly affected by inter-annual and microhabitat variability along the gradient, especially in the lowest edge. Despite the increasingly adverse environmental conditions, the plant showed no pollen limitation with elevation, which is attributed to the increase in autofertility levels (≥ 50% of reproductive output) and decrease in competition for pollinators at higher elevations. Plant fitness is, however, extremely reduced owing to the presence of non-native rabbits in the area (consuming more than 75% of the individuals in some localities), which in turn change plant trait-environment interactions along the gradient. Taken together, these findings indicate that the elevational variation found on plant performance results from the combined action of non-native rabbits with the microhabitat variability, exerting intricate ecological influences that threaten the survival of this violet species.

## Teaser

We studied the fitness of an endemic and vulnerable violet species along its altitudinal range (2,400 m - 3,500 m), and we found it to be mainly affected by inter-annual and microhabitat variability along the gradient, especially at the lowest edge. Despite the increasingly adverse environmental conditions, the plant showed no pollen limitation with elevation, which is attributed to the increase in selfing and decrease in competition for pollinators at higher elevations. Plant fitness was extremely reduced owing to the presence of non-native rabbits, which changed plant trait-environment interactions along the gradient, threatening the survival of this violet.

## Introduction

Mountain ecosystems worldwide provide sharp elevational gradients that allow testing ecological responses of biota to different abiotic influences across short distances (Dunne *et al.* 2003; Körner 2007), and therefore, are especially useful for understanding the response of plants to global change (Körner; 2007). Thus, alpine plant populations occurring at the lowest altitudinal distribution of the species should face especially harsh constraints on survival and performance mainly due to water scarcity and high temperatures, giving rise to species range shifts upward (Peñuelas and Boada 2003; Parmesan 2006). Moreover, the complex environmental heterogeneity present in alpine ecosystems may lead to phenotypic plasticity, and in some cases to genetic selection in favour of a particular reproductive behaviour in a specific environment and, hence, to local adaptation in plants (Rahn 1983; Körner 2003).

Reproductive efficiency is one of the major constraints in high-mountain ecosystems, as the environmental conditions in alpine habitats often reduce the possibilities of cross-pollination, affecting several fitness traits such as pollinator visitation rate (McCall and Primack 1992; Totland 1994) and fruit and seed set (Santandreu and Lloret 1999; Lundemo and Totland 2007; Ye *et al.* 2011). However, pollen limitation does not seem to differ overall between alpine and lowland species (García-camacho and Totland 2009), suggesting that alpine species may have compensatory mechanisms such as higher pollinator efficiency and extended flower longevity (Arroyo *et al.* 1985; Fabbro *et al.* 2004) to compensate for a lower visitation frequency in terms of their reproductive success. Furthermore, alpine plants show a great variability in germination behaviour, and may largely vary within a single species from one population to another (Urbanska and Schütz 1986; Giménez-Benavides et al. 2005).

On oceanic islands, high mountain areas represent one of the most isolated ecosystems on earth, which confers an outstanding uniqueness on their biota (Steinbauer *et al.* 2012). This biota frequently displays high endemicity (Steinbauer *et al.* 2016), usually well adapted to poor developed soils and to hydric, thermic or aeolian stresses, constituting islands within islands (MacArthur and Wilson 1967; Steinbauer *et al.* 2016). Therefore, high-mountain insular biota is particularly vulnerable to climate change and disturbance (Harter *et al.* 2015). The special vulnerability of this ‘sky island’ biota is mainly due to their small and isolated distributions, which prevent species migrating and escaping to a more favourable location (Fernández-Palacios *et al.* 2014). Consequently, rapid adaptation or high plasticity would be needed to cope ongoing climatic changes (Ohlemüller *et al.* 2008; Hampe and Jump 2011). Moreover, oceanic islands have relatively low species richness, simplified biotic community structures, and are characterized by having limited biotic exchange compared to mainland ecosystems (Whittaker and Fernández-Palacios 2007; Fordham and Brook 2010; Gilman *et al.* 2010). Hence, changes in the abundance of single members of functional groups (e.g., pollinators, herbivores) are likely to affect the structure and functional integrity of communities on oceanic islands more than in mainland areas. This is why the introduction of non-native species is one of the greatest threats to oceanic island ecosystems (Gillespie *et al.* 2008; Irl *et al.* 2014; Kueffer *et al.* 2014).

Introduced species are expected to interact with climate change (Walther *et al.* 2009; Mainka and Howard 2010), which will likely intensify the impacts on island ecosystems, increasing the need to effectively manage non-native species (Hellmann *et al.* 2008; Vorsino *et al.* 2014). Antagonistic interactions, such as grazing pressure by herbivores, can cause profound synergistic effects on climatically weakened native plant species (Gangoso *et al.* 2006; Krushelnycky 2014). These synergistic effects can be even worse in oceanic islands, where most plant species have either lost or never developed defense mechanisms against herbivory. Such impacts are likely to be greater than simple additive effects of single stressors (Brook *et al.* 2008; De Sassi *et al.* 2012) and, thus, can even have more dramatic consequences on the fragile ecosystems of summit oceanic areas.

Our general objective in this study was elucidating the factors affecting performance of an oceanic high-mountain endemic plant, *Viola cheiranthifolia* Humb. & Bonpl. Specifically, we asked the following questions: i) Does plant performance change along the pronounced elevational gradient (c. 1300 m), and are changes consistent between years? ii) Is the plant pollen-limited at high elevations, and does autofertility act as a compensatory mechanism? and iii) How does herbivory by rabbits (*Oryctolagus cuniculus* L.) influence plant performance along the elevational gradient? We expected that plant performance is limited at both edges of the altitudinal range, due to the warmer and dryer conditions (at the lowest edge) and to the harsh conditions for pollination (at the summit). We also expected to find pollen limitation at the summit due to possible lower pollinator visit rates, which could be compensated by higher selfing rates. Finally, we hypothesized that rabbits may be strongly altering the plant trait-environment interactions along the gradient, since they are known to cause pernicious effects on other endemic and threatened plant species of summit scrub systems of oceanic islands (Carqué-Álamo *et al.* 2004; Irl *et al.* 2012).

## Methods

### Study system

*Viola cheiranthifolia* is a high mountain dwarf chamaephytic plant, endemic to Tenerife. It is the most dominant and structuring species within the summit vegetation of El Teide. The largest populations are found around El Teide stratovolcano at elevations from c. 2400 m to c. 3700 m at the peak of El Teide, although some small populations occur at the highest points of the caldera (Guajara; 2715 m, Pasajirón; 2531 m). The plant grows on poor soils on cinder flats amongst the volcanic rubble, mixed with pumice stones at some localities. Its height usually ranges from 3 to 6 cm, and it has an oval-shaped hairy leaves. The species produces chasmogamous zygomorphic flowers mainly from late April to early July (although some flowers can be sometimes observed by mid-late winter). Flowers are found on 6–10 cm peduncles; they remain open for approximately 10 days, being white for the first 24–48 h after anthesis and becoming purple-violet afterwards. They have a short nectar spur (9 mm long) and produce a small amount of nectar. According to the *Viola* dispersal syndromes described by Beattie and Lyons (1975), *V. cheiranthifolia* presents typical diplochory, with the explosive ejection of seeds, and a small caruncle (shrunk elaiosome) permitting post-dispersal by ants (myrmechory). Despite this, no secondary dispersal by ants has ever been recorded.

### Research site

**Figure 1.**
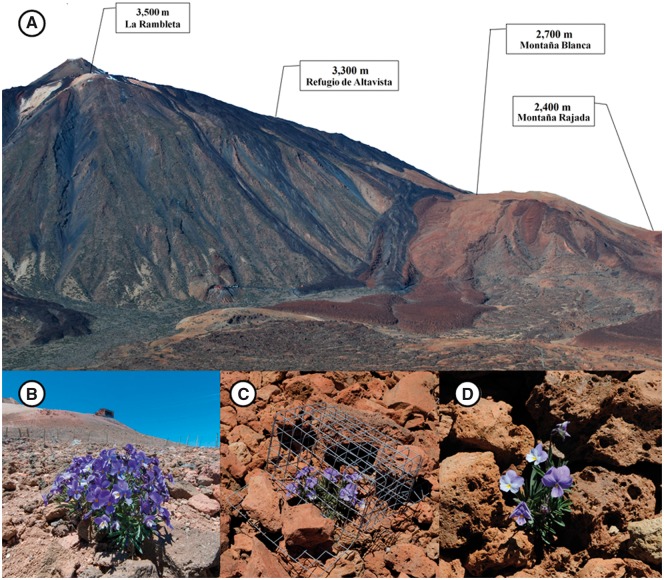
1A; study sites selected on the SE slope of the Teide stratovolcano. 1B; Plant located inside the herbivore fence exclusion at 3500 m. 1C; Plant with the individual exclusion cage. 1D; Image of a whole non-protected plant, with presence of pumice, which protects individuals and maintains soil moisture.

Fieldwork was conducted during four years (2012–2015) at El Teide National Park (N.P). El Teide stratovolcano (3718 m; 28º16’15’’N 16º38’21’’O), like many tropical and sub-tropical alpine landscapes, presents xeric, arid environments (Leuschner 2000). The peak of El Teide (La Rambleta, hereafter) has a mean annual precipitation of 146 mm and a mean temperature of 4.2ºC (period 1989–1992, ICONA: in Gieger and Leuschner 2004). We set an elevational gradient along four different localities of *V. cheiranthifolia*, separated between 200 and 600 m from each other: Montaña Rajada (2400 m), Montaña Blanca (2700 m), Refugio de Altavista (3300 m), and La Rambleta (3500 m) (Fig. 1A). Temperature and edaphic (soil moisture and organic matter) data were gathered at each site to control for differential abiotic conditions along the gradient (Table 1). All study sites were located on the SE face of the stratovolcano (the only slope that covers the entire elevational distribution of the plant) in order to more accurately control for the elevational effect (Fig. 1A). Plant density varied depending on microsite availability in each site; overall, however, plant density in our study sites were rather similar, ranging from 0.01 to 0.03 individuals m^−2^.

**Table 1.** Temperature and edaphic data at each site. Temperature (T) was measured with a temperature data logger during a whole year (2014–15). Soil moisture was measured with a soil moisture sensor with 7–11 replications per site. We did not get climatic data from the lowest site (2400 m), instead we present data of a data logger located at 2153 m. O.M. (organic matter).

Site	Coordinates	Altitude	T. Max. (ºC)	T. Min. (ºC)	Mean (ºC)	Max. Diurnal oscillation (ºC)	O.M. (%)	Soil moisture (%)
Cañadas del Teide	28 13.455 N	2153 m	30.78	−5.87	11.69	22.25	(–)	(–)
	16 37455 W							
Montaña Blanca	28 16.195 N	2732 m	34.05	−6.86	10.85	29.88	1.76	11.63
	16 36.938 W							
Refugio de Altavista	28 16.469 N	3296 m	33.24	−10.91	8.34	35.11	0.02	9.53
	16 37.789 W							
La Rambleta	28 16.490 N	3518 m	24.96	−13.03	5.02	23.19	0.03	12.98
	16 38.768 W							

### Effect of elevation on plant performance

Phenology, plant size, and flower display were studied along the main elevational gradient in 2012 and 2013 on a total of 329 and 204 individually marked plants, respectively. Since early April, we began marking reproductive individuals until we reached a minimum of 50 per locality. During 2013, aside from sprouted individuals marked in 2012, we added some new reproductive individuals to reach this number. To assess differences in flowering and fruiting phenology, each locality was visited once every 3–7 days until August. In each visit, we recorded the number of buds and flowers at different stages of anthesis, as well as the number of fruits produced. Plant size (maximum length and width of each individual) was also measured to account for its potential effect on reproductive success.

To quantify the reproductive success along the elevational gradient during 2012 and 2013, differences in fruit set (number of flowers setting fruits) and number of seeds per capsule were compared using 428 control flowers in a total of 106 marked individuals. Seed production was quantified by individually bagging 245 fruits (in a total of 97 individuals) before ripening and counting the number of seeds after fruits opened. All marked individuals from the lowest elevation (2400 m) had to be excluded from the reproductive success analyses because the number of reproductive individuals was very low (only 7 individuals in 2012) or even nil (in 2013).

To evaluate the level of autofertility (capacity to produce seeds by spontaneous self-pollination; Eckert *et al.* 2010), half of the flower buds available per individual were bagged before flower anthesis with a mesh-cloth that excluded floral visitors. Control flowers (open-pollination) from the same individuals were also marked and left un-bagged. Due to the high flower consumption by herbivores, we finally also used flowers from non-treated individuals as control. Autofertility was quantified in 2012 and 2013 in a total of 126 bagged flowers from 53 individuals from three different localities at 2700, 3300 and 3500 m (20, 15 and 18 individuals, respectively).

To examine whether the extent of pollen limitation differed among localities, we hand-pollinated half of the flowers available per plant, leaving the other half as controls. Pollen addition (PA) was accomplished by brushing pollen-laden anthers of about 5–10 plants from another population. This procedure was repeated two days during stigma receptivity. Pollen limitation was quantified in two consecutive years (2014 and 2015) in a total of 49 individuals from three different localities (23 individuals at 2700 meters in 68 PA flowers, 15 individuals at 3300 m in 29 PA flowers, and 11 individuals at 3500 m in 44 PA flowers).

Floral visitors were quantified and compared along the elevational gradient during 2014 and 2015. We conducted a total of 182 censuses of 15 min (32 censuses at 2400 m, 57 at 2700 m, 47 at 3300 m and 46 at 3500 m). We measured flower visitation rate as the number of visits per hour per flower. Censuses were carried out in no-windy days, mostly from 10:00 to 16:00, avoiding the beginning and the end of the day, with usually low temperatures and little insect activity in this high mountain environment. Only visitors touching the reproductive parts of the flowers were considered; these were either identified in the field or collected for later identification in the laboratory.

Seed germination was assessed along the elevational gradient in 2012. Of the 1224 seeds collected, 44.5 % were stored in the dark at room temperature and the remaining 55.5 % underwent a stratification treatment (2-month below 5ºC). Seeds were buried in late February 2013, in a greenhouse located within the N.P at 2000 m. Moreover, during 2013 we evaluated the seed viability by means of the tetrazolium-chloride test (TTC methods in Marrero *et al.* 2007) in a total of 3824 control seeds.

### Effect of herbivore exclusion on plant performance

The effect of herbivory upon *V. cheiranthifolia* was assessed by: (1) recording, at all localities, the proportion of individuals consumed (totally or partially) by rabbits (clipping off sprigs, stems and buds) and the number of consumed flowers per individual in 2012 and 2013 (besides rabbits, lizards and grasshoppers were occasionally observed consuming flowers); (2) comparing fitness traits (size, floral display, fruit and seed set) in protected individuals (by individual exclusion cages; Fig. 1C) during a two year period (2014 and 2015) among elevational populations; and (3) comparing plant density, fitness traits, autofertility, and pollen limitation (with the same methods described above) between 25 individuals inside and 35 outside a rabbit exclusion fence during 2013. This herbivore exclusion (20 × 20 m, located at 3500 m; Fig. 1B) was set up by the N.P. in 2009. Individuals outside the exclosure were used as controls, and were located between 5 and 90 m from the fence. The effect of herbivore exclusion on seed viability was assessed by comparing a total of 1511 seeds from 25 individuals within the exclosure with 677 seeds from 19 individuals outside it.

Finally, we wanted to determine whether herbivore exclusion affected flower visitation rate. For this, we carried out pollinator censuses inside and outside the exclosure. A total of 49 and 46 censuses of 15 min were accumulated during 2014 and 2015 within and outside the exclosure, respectively; individuals selected for censuses outside were at a minimum distance of 5 m and a maximum of 100 m from the fence.

### Data analysis and statistics

To test for differences in individual size among localities along the elevational gradient and between years, plant area was estimated by using maximum perpendicular diameters of the whole plant, subsequently log_10_-transformed to meet the assumptions of normality. We used General Linear Mixed Models (GLMM; package lm4 in R) for the analyses of between-year and among-population variations in plant size, flowering probability, floral display, fruit set, seeds per capsule, pollination rate, and herbivore rates. We included elevation, year, and the elevation x year interaction as fixed factors, plant individual as a random effect, and plant size as covariate; for some variables, the interaction between plant size and elevation was also included in the model. Seed viability was compared in 2013 among elevations including plant size as covariate and individual plant as a random effect. Resprouting probability (measured only in 2013) was compared among elevations using General Linerar Models.

The levels of autofertility and pollen limitation in *V. cheiranthifolia* were compared across localities by means of GLMM, with fruit set and seed set as dependent variables, pollination treatment and elevation as fixed factors, and individual plant and year as random effects. The germination rate was compared across localities using the Kolmogorov-Smirnov test.

To test for plant responses to elevation after one and two years of individual protections we used size, floral display, seed set and fruit set as dependent variables, elevation, year and the elevation x year interaction as fixed factors, and plant individual as random effect. Finally, to test for the influence of the four-year herbivore exclusion on plant fitness, we compared those four variables between excluded and non-excluded plants.

We used error distribution and link functions that best fit the data: (1) binomial distribution for fruit set, seed viability, and probability of flowering and resprouting (2) a Gaussian distribution for plant size, (3) a Poisson distribution for floral display and seeds per capsule, and (4) a gamma distribution and inverse link function for pollination rate (visitation rate + 1, in order to avoid zeros in the response variable). When seeds per capsule showed overdispersion (this occurred when comparing elevations), we performed a negative binomial model (Zuur *et al.* 2009). All predictor variables showed VIF values smaller than three and were, therefore, included in the analyses (Zuur *et al.* 2009). We used the Akaike information criterion to select the best models with the package MuMIn 1.15.6, with ΔAIC values > 2 retained as indicators of a significantly improved model fit. All statistical analyses were performed with the R package, v. 3.1.

## Results

### Effect of elevation on plant performance

Floral buds of *V. cheiranthifolia* showed an abundance peak between late April and mid-May both years that phenology was monitored **[see****Supporting Information—Fig. S1****]**. Open flowers were available from the first week of April until early July; the highest bloom occurring by mid-end of May. The fruiting peak was by mid-end of June, although some individuals showed fruits already by mid-late April whereas others fruited during late August. Plants at the highest locality (3500 m) flowered and fruited about ten days later than plants at lower elevations at 2013, with no differences in the non-snowy 2012 **[see****Supporting Information—Fig. S1****]**.

**Figure 2.**
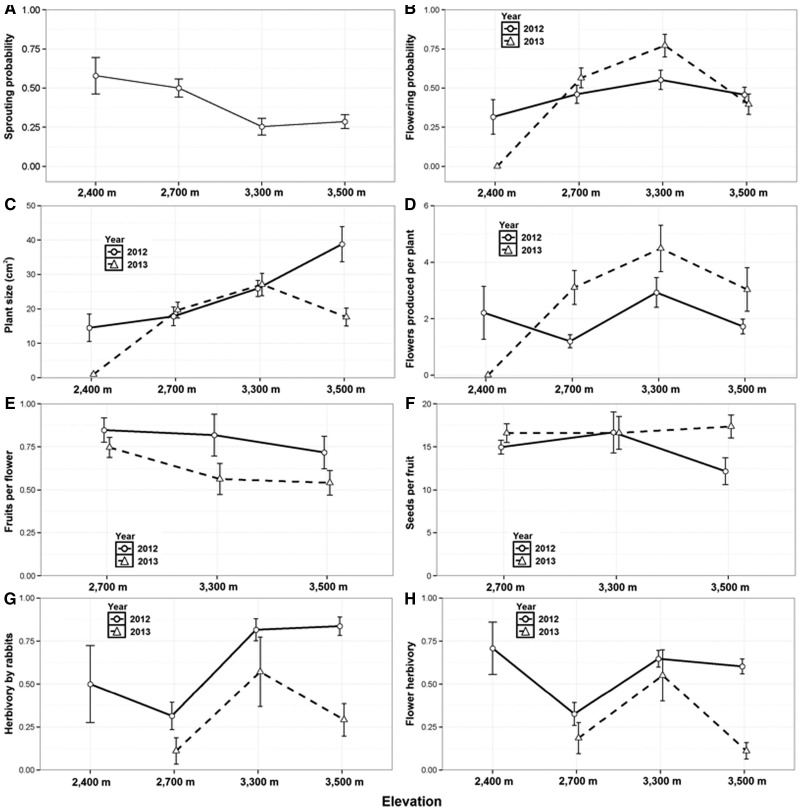
Plant traits studied along the elevational gradient during 2012 and 2013. 2A; Sprouting probability represents the percentage of individuals marked in 2012 that sprouted in 2013. Points represent means and vertical bars represent standard errors.

The proportion of individuals that resprouted in 2013 after wintertime was higher at low-sites (2400 m and 2700 m) than at high-sites (3300 m and 3500 m) (Z-value  =  −2, *P *< 0.05; Table 2A;Fig. 2A). We did not detect significant effects of plant size on resprouting probability (Table 2A). Plant size varied among elevations but not consistently between years (Table 2A): in 2012, plant size increased with elevation (*F*_3,266 _ = _ _14.58, *P* < 0.001), whereas in 2013 it was greatly reduced at the two extremes of the distribution, though mainly at 2,400 m (Fig. 2C). Flowering probability (Fig. 2B), by contrast, did mainly depend on plant size, the largest plants being more likely to bloom (Table 2A); this was consistent across localities, but with different slopes. At 2700 m and 3300 m flowering probability increased more strongly with plant size (slope: 0.16 ± 0.03, 0.21 ± 0.04, *P* < 0.0001, respectively) than at the extremes of the plant distribution, at 2400 m and especially at 3500 m (slope: 0.14 ± 0.07, 0.01 ± 0.00, *P* ≤ 0.05, respectively). Larger plants produced also more flowers in all localities, but this increase with size was smaller in the highest elevation (slope: 0.01 ± 0.002, *P* < 0.0001, Table 2A); in 2013, plants produced more flowers than in 2012, except at 2400 m where no plants flowered (Fig. 2D).

**Table 2.** Results of GLMM for (a) response variables measured in non-protected individuals (2012–2013), (B) autofertility (2012–13) and pollen addition (2014–15) treatments and (C) response variables measured in caged plants along the altitudinal gradient (2014–15). Rows: factors included in each model. Columns: the response variables. We provide the sign of the effect for the continous predictors, the chi-squared value and the significance level: non significant (n.s.) *P ≥ *0.05, *P < 0.05, **P < 0.01, ***P > 0.001.

A) Factors	Resprouting	Flowering	Plant size	Floral display	Fruit set	Seed set	Herbivory	Florivory
Altitude	14.36**	(–)	(–)	(–)	1.88 n.s.	0.03 n.s.	33.53***	54.71***
Year	(–)	(–)	(–)	11.1***	9.62**	2.06 n.s.	23.7***	81.03***
Altitude x Year	(–)	7.03 n.s.	12.08**	(–)	(–)	(–)	(–)	(–)
Size	(–)	(–)	(–)	(–)	(–)	+10.79**	(–)	(–)
Altitude x Size	(–)	141.5***	(–)	201.34***	19.98***	(–)	(–)	(–)

B) Factors	Fruit set	Seed set		Fruit set	Seed set
*Altitude*	(–)	(–)	*Altitude*	6.39 n.s.	0.71 n.s.
*Autofertility*	(–)	(–)	*Pollen addition (PA)*	6.20*	0.97 n.s.
*Altitude x Autofertility*	11.17**	15.51**	*Altitude x PA*	(–)	(–)
*Herbivore exclusion (HE)*	(–)	30.03***	*Herbivore exclusion (HE)*	4.31*	1.55 n.s.
*Autofertility*	(–)	99.64***	*Pollen addition (PA)*	0.08 n.s.	0 n.s.
*HE x Autofertility*	21.02***	(–)	*HE x PA*	(–)	(–)

C) Factors	Fruit set	Seed set	Fruit set	Seed set
*Altitude*	(–)	(–)	8.63*	3.06 n.s.
*Year*	(–)	(–)	(–)	1.31 n.s.
*Altitude x Year*	6.42*	7.77*	(–)	(–)
*Cages – Fence Exclusion*	3.26 n.s.	5.01*	0.75 n.s.	2.1 n.s.
*Year*	3.36 n.s.	129.16 n.s.	(–)	8.17**


Fruit set was higher in 2012 than 2013 but did not vary among elevations either of the two years (Table 2, Fig. 2E). Interestingly, while at 2700 m and 3300 m fruit set increased significantly with size (slope: 0.06 ± 0.02), this relationship was negative at 3500 m (slope: − 0.03 ± 0.01). Number of seeds per capsule, on the other hand, only differed between years at the highest elevation, and larger plants consistently produced more seeds per fruit in all localities (Table 2A, Fig. 2F).

Seed viability varied significantly among elevations (*χ*^2^_2 _ = _ _17.66, *P* < 0.001): seeds from 2700 m were less viable (c. 80%) than those at either 3300 m or 3500 m (≥ 90%). There was no plant size effect on seed viability (*χ*^2^_1 _ = _ _2.6, *P*  =  0.1).

**Figure 3.**
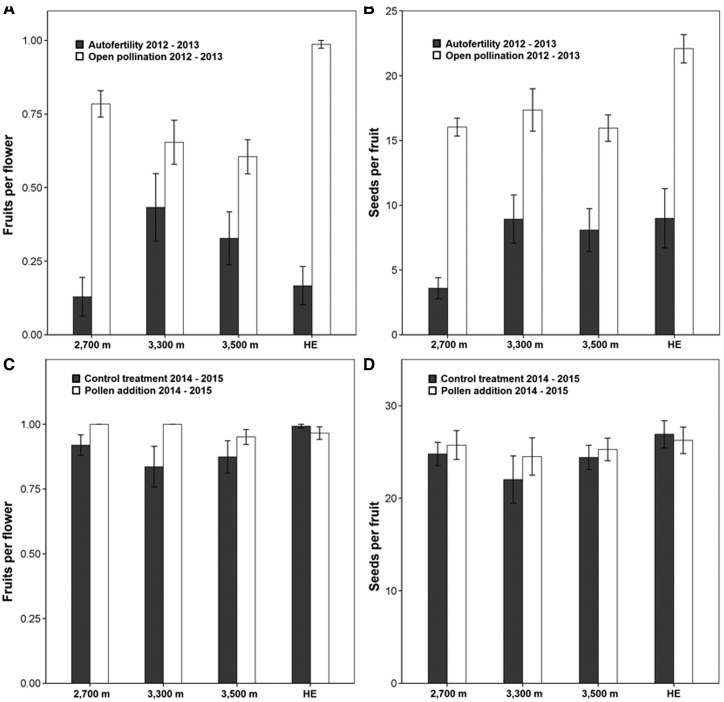
Autofertility and pollen addition treatments accounting for fruit set and seed set. Treatments were performed in different pairs of years (2012–13 and 2014–15) due to the low flower display per plant. 3AB; Autofertility treatment in the herbivore fence exclusion was performed only during 2013. Bars represent means and vertical bars represent standard errors. HE indicates herbivore exclusion.

Both fruit set and seeds per capsule were significantly higher in the open-pollination than in the autofertility treatment (*P* < 0.0001; Fig. 3A and B). Autofertility varied across elevations, being higher at 3300 m and 3500 m than at 2700 m, and this was observed both for fruit set and seeds per capsule (Table 2B;Fig. 3A and B). Fruit set was consistently over 75% in all localities during 2014 and 2015, and was only slightly pollen limited. Number of seeds per capsule, on the other hand, showed no pollen limitation at any elevation (Table 2B;Fig. 3C and D). Neither plant size nor floral display was included in any of the best models.

**Figure 4.**
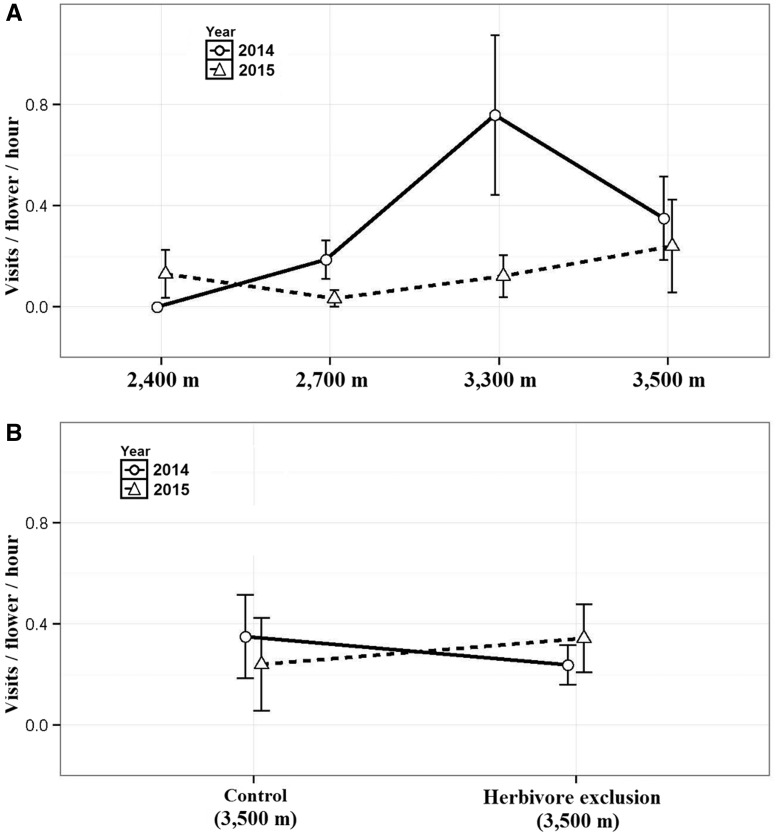
Flower visitation rate among elevational localities and between rabbit-excluded and non-excluded plants (2014–2015). Points represent means and vertical bars represent standard errors.

The number of plant species and floral visitors in the Teide’s alpine community decreased along the elevational gradient during 2014 and 2015 (Traveset, unpub. data); however, in the case of *V. cheiranthifolia* the number of interactions increased with elevation during those years (Table 3). This violet was visited by a total of 19 native insect species and the non-native *Apis mellifera* (Table 3). Of these, the most dominant species were the bee *Anthophora alluaudi* and the honeybee *Apis mellifera*. Mean visitation rate during the two years was 0.28 visits per flower per hour. Assuming a 6-h activity period for pollinators and that flower duration is c. 10 days, flowers received on average ∼16.8 visits during their entire lifespan. Such visitation rate varied among elevations (*F*_3, 181__* *_ = _* *_5.14, *P*  =  0.001; Fig. 4A), tending to be higher at 3300 m than at 2700 m, and being higher in 2014 than in 2015 overall (*F*_3, 181__* *_ = _* *_4.53, *P* < 0.05; Fig. 4A).

**Table 3.** Floral visitors of *V. Cheiranthifolia* observed at each elevational site during 2014–15.

Order	Species	2400 m	2700 m	3300 m	3500 m
**Hymenoptera**					
Anthophoridae	*Amegilla quadrifasciata*		Χ		
Anthophoridae	*Antophora alluaudi*	Χ	Χ		Χ
Apidae	*Apis mellifera*		Χ	Χ	Χ
Encyrtidae	*Homalotylus sp.*				Χ
Eulophidae	*Elasmus sp.*				Χ
Megachilidae	*Megachile canariensis*				Χ
Pteromalidae	*Systasis sp.*				Χ
**Lepidoptera**					
Lycaenidae	*Cyclyrius webbianus*			Χ	Χ
Pieridae	*Pieris rapae*		Χ		
Pieridae	*Euchloe belemia*		Χ		
Sphingidae	*Macroglossum stellatarum*				Χ
**Diptera**					
Phoridae	*Phoridae sp.*			Χ	
Sirfidae	*Scaeva albomaculata*			Χ	Χ
Tachinidae	*Linnaemya soror*			Χ	
Tachinidae	*Pseudogonia fasciata*		Χ		
**Coleoptera**					
Melyridae	*Melyrosoma hirtum*			Χ	Χ
Nitidulidae	*Meligethes varicollis*			Χ	
Buprestidae	*Acmaeodera cisti*		Χ		
**Hemiptera**					
Lygaeidae	*Nysius cymoides*			Χ	
Anthocoridae	*Orius laevigatus*			Χ	
**TOTAL**		**1**	**7**	**9**	**10**

The germination rate was extremely low, with only 3.82% of emergence of stratified seeds and 2.92% of non-stratified seeds. Except for seeds collected at 3500 m, which showed significantly higher germination rate when stratified (*χ*^2^_1__* *_ = _* *_4.71, *P *< 0.05), there were no differences between stratified and non-stratified seeds in other localities. Moreover, a significantly higher germination rate was encountered only when comparing seeds at 3500 m with those at 2700 m (*χ*^2^_1__* *_ = _* *_6.75, *P* < 0.01).

### Effect of herbivore exclusion on plant performance

A high fraction of the total marked individuals underwent herbivory in all localities, with a mean per locality ranging from 31 to 83% in 2012 and from 11 to 57% in 2013. Great differences were observed both in the proportion of consumed plants and flowers among elevations and between years (Table 2C). We did not observe an upward trend with elevation in both herbivory and florivory, but both were consistently low at 2700 m; Fig. 2G and H).

The density of violets was higher inside than outside the exclosure (9.05 individuals m^−2^*vs*. 0.15 individuals m^−2^), and most reproductive parameters differed significantly between excluded and non-excluded individuals. Inside the exclosure, plants were larger (*F*_1,52__* *_ = _* *_107.3, *P* < 0.0001), showed greater floral display (*χ*^2^_1 _ = _ _96.08, *P* < 0.0001 with size as covariate; *χ*^2^_1_ = 686, *P *< 0.0001), higher fruit set (*χ*^2^_1 _ = _ _26.69, *P* < 0.0001) and seeds per capsule (*χ*^2^_1 _ = _ _32.63, *P* < 0.0001), and higher seed viability (*χ*^2^_1_= 14.1, *P* < 0.001) than controls. Seed viability was higher inside the exclosure (94 ± 0.07%) than outside it (90 ± 0.16%). Pollinator visitation rate per flower did not differ between either plant inside and outside the exclosure (*F*_1,94__* *_ = _* *_0.04, *P * =  0.84; Fig. 4B) or between years (*F*_1,94__* *_ = _* *_0.0001, *P * =  0.99; Fig. 4B).

Bagged flowers within the exclosure produced fewer fruits per flower – but not fewer seeds per fruit – than bagged flowers outside the exclosure (Table 2B, Fig. 3A and B). In contrast, a consistently higher fruit and seeds per capsule was found in open-pollinated flowers within the exclosure than outside it. Moreover, no pollen-limitation was detected in either excluded or non-excluded individuals (Table 2B, Fig. 3C and D).

**Figure 5.**
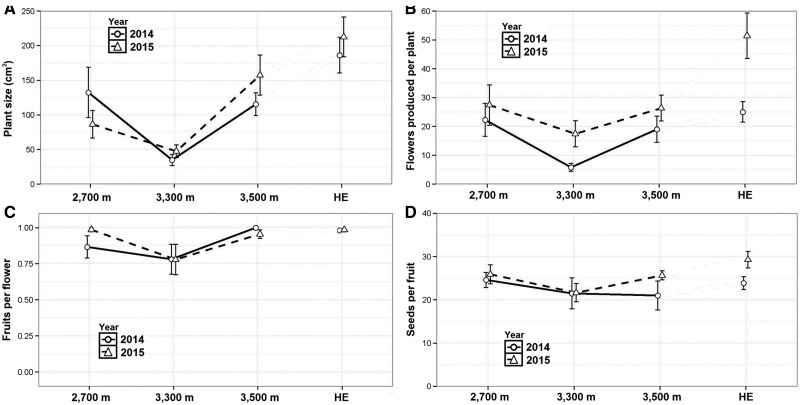
Plant traits of rabbit-excluded individuals along the elevational gradient and in the 3500 m fence exclusion during 2014 and 2015. Points represent means and vertical bars represent standard errors. HE indicates herbivore exclusion.

Finally, when examining plant size and reproductive traits along the elevational gradient after either one or two years of individual rabbit exclusions, we found that plants at 3300 m were smaller, produced less flowers and had a lower fruit set than those at the other localities (Z-value > −2, *P* < 0.05, in all cases; Fig. 5A, B and C). However, we found an interaction with elevation for size and floral display (Table 2C). Seeds per capsule in excluded individuals showed no significant differences among localities (Table 2C, Fig. 5D). When plants within the exclosure were compared with those excluded either one or two years at 3500 m, all differences in plant size and reproductive fitness disappeared, except for a floral display, suggesting that plants respond rapidly to herbivore exclusion (Table 2C, Fig. 5).

## Discussion

The environmental variability found along the gradient showed a consistent trend only in mean temperature (Table 1), with differences in fraction of organic matter and in soil moisture depending on small-scale heterogeneity. The performance of *V. cheiranthifolia* showed to be especially constrained at the lowest elevation (2300 m), with a very low plant size and floral display, and with practically no individuals producing seeds, probably due to the warmer and dryer conditions at this site in the two dry years (2012 and 2013). Hence, it seems plausible that this endemism could be suffering an upward shift of its low edge limit under the effect of climate change (Klanderud and Birks 2003; Parmesan 2006). This, together with the strong deleterious effects of non-native rabbits observed on its fitness and reproduction reveals a very uncertain future for this unique endemic plant.

### Effect of elevation on plant performance

The annual variability in blooming suggests that it is mainly controlled by snow conditions or soil moisture, and not only by temperature and photoperiod as found in other studies (Walker *et al.* 1995; Keller and Körner 2003; Giménez-Benavides *et al.* 2007). Regarding plant regrowth, we found it to be lower at higher elevations, which we attribute to the fact that the severity and frequency of freezing temperatures significantly increase with elevation (Neuner 2014). The type of soil (pumice stones, Fig. 1D) of the lower localities retains water and might thus provide safe sites for plant development (Pérez 2009) leading to higher plant regrowth there.

Plant size increased with elevation in the unusual snowless 2012, which is not concordant with the growth-limitation hypothesis (Körner 1998). In 2013, however, plant size decreased at the two extremes of the elevational distribution, especially at the lowest elevation. The small plant size observed in both dry years (2012–13; Izaña Meteorological Station; www.ogimet.com) suggests that the species might be currently contracting its distribution due to the increasing warmer and dryer conditions, as found for other species (Engler *et al.* 2011; Gottfried et al. 2012). During snowy years, on the other hand, plants at the lowest elevation can take advantage of longer snow-free period (Palacios *et al.* 2003) and increase their size (we did observe large flowering individuals at this site in snowy years), despite this rapid growth might imply greater weakness at maturity and lower survival (Giménez-Benavides et al. 2006). The presence of small snowfalls during the winter 2012–13, with snow cover maintained longer at the upper site, might explain the decrease in size observed at 3500 m in 2013. *Viola cheiranthifolia* showed a high size-dependence at several stages of the reproductive process; thus, smaller plants were less likely to flower and produced fewer flowers, as reported for other species (Ollerton and Lack 1998; Méndez and Karlsson 2004). The tiny individuals at the lowest elevation (2400 m) actually produced no flowers at all in 2013. At 3500 m, larger plants were not as likely to flower and did not produce as many flowers as expected compared to plants at lower elevations, probably due to the unfavourable environmental conditions at the summit area (snow cover). Temperature is known to indirectly affect nutrient availability at high elevation (Körner 2003), whereas water availability is known to affect it at low elevation which could thus explain, at least partly, our findings. In short, plant size, flowering probability, and floral display of *V. cheiranthifolia* seem to vary on a spatiotemporal scale, being influenced by environmental conditions at each elevation, mainly snowfall regime which determines soil moisture and temperature.

Overall, contrary to our expectations, neither fruit set nor seeds per capsule varied with elevation. In spite of the assumption of scarcity of pollinators at alpine areas, literature shows no consistent trend of plant fitness along elevational gradients, with wide interspecific differences as well as temporal variation within species (Seguí *et al.* meta-analysis in prep.). Fruit set of *V. cheiranthifolia* varied between years, as reported for other alpine species such as *Silene ciliata* (Giménez-Benavides *et al.* 2007) and *Armeria caespitosa* (García-Camacho and Escudero 2009). Number of seeds per capsule of *V. cheiranthifolia* only differed between years at 3500 m; the lower values in the snowless 2012 are possibly due to the lower water availability compared to 2013, besides also a higher herbivory the former year (Fig. 2G).

The environmental conditions in alpine habitats may reduce the possibilities of cross-pollination and are believed to represent important driving forces in the evolution of self-pollination at high elevations (Molau 1993; Totland 2001; Kalisz *et al.* 2004). *V. cheiranthifolia* showed a high (c. 50%) autofertility level, especially at the two highest localities. This increase in autofertility level with elevation is consistent with other studies that have reported enhanced self-compatibility mechanisms at greater elevations (Medan *et al.* 2002; Arroyo *et al.* 2006). In addition, although the scarce evidence available from the literature shows that pollinator abundance is lower at high elevations (Cruden 1972; Totland 1994; Medan *et al.* 2002), we observed no increase in pollen limitation with elevation. This agrees with the findings of García-camacho and Totland (2009) who reported that ovule fertilization is not particularly limiting in highland *vs.* lowland sites and attribute this to plant compensatory mechanisms to combat the scarcity of pollinators. Flower visitation rates showed to vary among localities and in time, what is often attributed to variable climatic conditions (Totland 1994; Herrera 1995), but were not found to decrease with elevation as expected. At lower elevations, *V. cheiranthifolia* coexists with a large variety of entomophilous plants whereas at 3500 m this violet is the only plant in the pollination community; this lack of interspecific competition for pollinators at high elevations might thus lead to a greater generalization level.

The fact that seed viability was positively associated with elevation, with lower values at 2700 m than at 3300 or 3500 m, is in line with other studies (Marcora *et al.* 2008; Milla *et al.* 2009) and might be due to different reasons. First, the abundance of heterospecific pollen can be expected to be higher at lower elevations due to the presence of other flowering plants, potentially affecting seed set and viability (Morales and Traveset 2008). Second, seeds at higher elevations might be expected to ripen more slowly, owing to the lower temperatures and longer drought periods than those in the lowest localities, which might positively influence embryo viability and germination (Fenner and Thompson 2005). Despite the relatively high seed viability found, the seeds of *V. cheiranthifolia* showed a very low germination rate. This is possibly related to the ability to produce an abundant seed bank capable of dormancy that guarantees the long-term reproductive success of the species, even when climatic conditions are adverse or pollinators are scarce (Pannell and Barrett 1998).

### Impact of invasive rabbits on plant performance

Herbivores can have strong deleterious effects on plant growth, reproduction and even survival (Crawley 1989; Marquis 1992; Strauss and Zangerl 2002). These deleterious effects have a disproportionally greater impact on island endemic plants such *V. cheiranthifolia*, because these are expected to have lost, through the evolutionary process, the defense mechanisms and strategies from their ancestors (Burger and Gochfeld 1994; Atkinson 2001; Courchamp *et al.* 2003).

For this reason, herbivore pressure by non-native rabbits upon *V. cheiranthifolia* was huge in almost all elevational localities, affecting even more than 75% of the individuals in some of them. Pressure was variable among elevations and between years, probably due to the changes in rabbit density and population dynamics between sites and years. Very few studies have demonstrated spatial variation in the magnitude of herbivory along elevational gradients (Galen 1990; Bruelheide and Scheidel 1999), and as far as we know, this is the first study that examines so in a high-mountain oceanic ecosystem. Moreover, herbivores strongly altered many of the studied plant traits. After excluding rabbits for two years (2014–15), the effect of elevation on plant size, floral display, and fruit set changed completely from that previously observed in the non-excluded plants, highlighting that the current state that we observe in this endemism is far from what would be expected without the presence of rabbits. Consistently, both in 2014 and 2015, these three performance traits showed the lowest values at 3300 m but were similar at 2700 m and 3500 m. One reason for this may be that the population at 3300 m had the lowest soil moisture and the highest annual and daily temperature oscillation (Table 1); by contrast, soil moisture at 3500 m is maintained high after snow melt, whereas at 2700 m, the dominant substrate is pumice grits, what also maintains moisture for long periods (Fig. 1C and D).

Rabbits, in particular, are among the 100 most invasive species (Lowe *et al.* 2000), and are virtually present on all major islands of the world, where they have devastating effects (Courchamp *et al.* 2000). Although further studies are needed to quantify to what extent herbivory upon *V. cheiranthifolia* is altering population dynamics in the long-term, it is very revealing that, in all localities, plant performance is much higher when herbivores are excluded. Moreover, herbivory pressure might be affecting the reproductive ecology of the species, as we found a lower autofertility level at the rabbit exclosure. In this sense, and in the light of what is occurring within the exclosure, it is clear that rabbits considerably alter and diminish the distribution, abundance and fitness of this summit endemic violet.

Finally, herbivory can also alter mutualistic interactions, such as those between plants and their pollinators. Leaf damage by herbivores can decrease the overall plant attractiveness to pollinators (Lehtilä and Strauss 1997; McCall and Irwin 2006), greatly reducing the opportunity for pollinators to select between plants (Herrera 2000; Herrera *et al.* 2002). One reason for the increase in fruit set observed when rabbits are excluded could be an individual higher floral display and therefore higher attractiveness to pollinators. No differences, however, were detected in flower visitation rate between protected and unprotected plants in any of the two years of intensive censuses (2014–15), which suggests some kind of resource limitation influencing fruit and seed set. Plants excluded from herbivores can grow larger, and possibly be less resource-limited as they may be capable of extracting more resources from the soil and accumulate rhizome reserves than small plants (in fact, we only observed rhizome reserves in plants from the exclosure; pers. obs.).

## Conclusions

In short, our findings demonstrate that drivers associated with elevation are not necessarily limiting plant reproductive success, with no differences in pollen limitation and a higher self-pollination rate as a compensatory mechanism in the highest localities. *V. cheiranthifolia* showed to be mainly affected by local conditions related to environmental heterogeneity, especially soil moisture (influenced by snow duration and soil type) and co-occurring plant species, at each site and year, and mainly by the interactive effect of non-native rabbits, which appear to be altering plant responses along the gradient. Rabbits are acting as ecological engineers, intensely altering this isolated, fragile, and exclusive island ecosystem, in which the violet is the most dominant species in the community above a certain elevation. We argue that only by urgently minimizing the effects of invasive herbivores by effective control campaigns can this ecosystem be restored to the most natural state possible.

## Sources of Funding 

Our work was funded by a project of the “Organismo Autónomo de Parques Nacionales” (785/2012). Jaume Seguí was supported by the Graduate Fellowship Program co-funded by the European Social Fund (ESF) and the Government of the Balearic Islands (Conselleria d'Educació, Cultura i Universitats), and Marta López-Darias by a JAE Doc programme of the Spanish National Research Council.

## Contributions by the Authors

AT and MN conceived and designed the study. JS, MLD, and AJP conducted field work. JS and ML analyzed the data. JS and AT led the writing with valuables contributions from the rest of authors.

## Conflict of Interest Statement

None declared.

## Supplementary Material

Supplementary Data

## Supporting Information

The following additional information is available in the online version of this article—

**Figure S1**. Percentage of buds, flowers and fruits produced during the flowering season of *Viola cheiranthifolia* during 2012 and 2013 at each elevation. Round unfilled marks reflect inferred values obtained by averaging adjacent dates, due to the impossibility of visiting that particular locality on that date.
