# Supplementary material for: Species–environment interactions changed by introduced herbivores in an oceanic high-mountain ecosystem
Source: AoB Plants. 2017 Jan 5;9(1):plw091. doi: 10.1093/aobpla/plw091 (PMC5497022; doi:10.1093/aobpla/plw091)
Supplement: Supplementary Data [file plw091_Supp.docx]

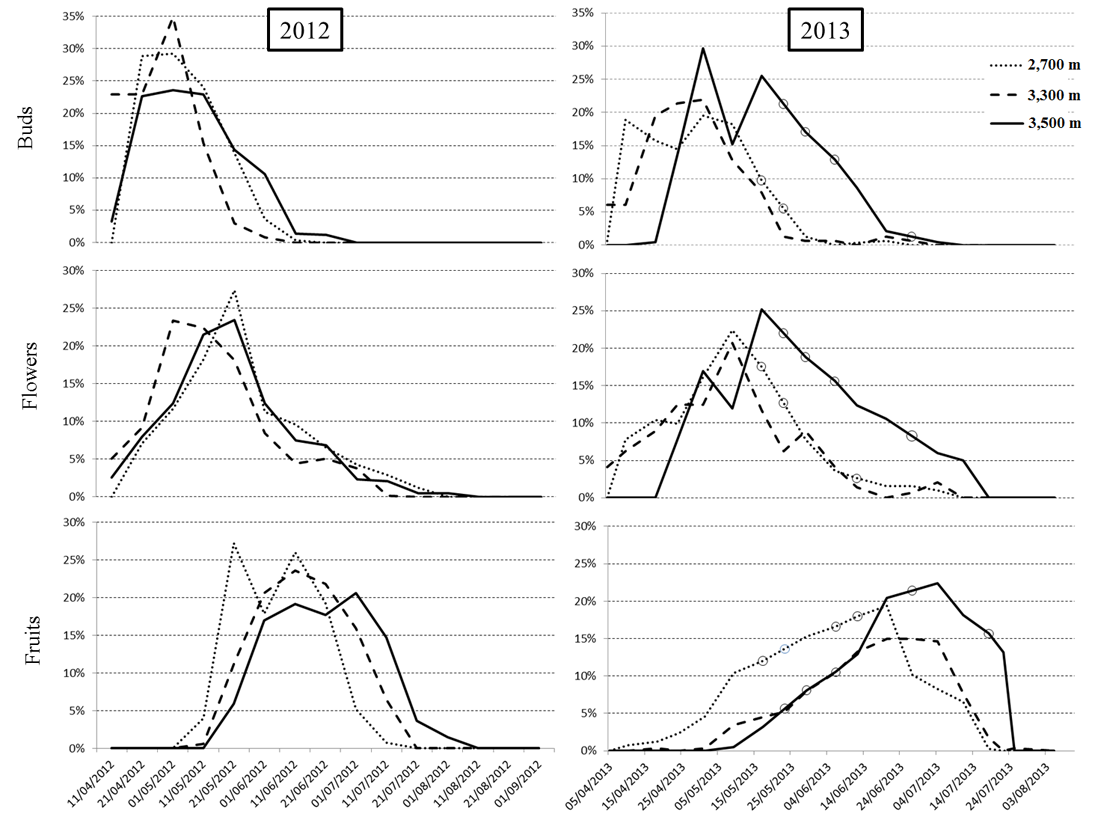


Figure S1. Percentage of buds, flowers and fruits produced during the flowering season of *Viola cheiranthifolia* during 2012 and 2013 at each elevation. Round unfilled marks reflect inferred values obtained by averaging adjacent dates, due to the impossibility of visiting that particular locality on that date.
